# *FIR* haplodeficiency promotes splicing to pyruvate kinase M2 in mice thymic lymphoma tissues revealed by six-plex tandem mass tag quantitative proteomic analysis

**DOI:** 10.18632/oncotarget.19061

**Published:** 2017-07-07

**Authors:** Asako Kimura, Kouichi Kitamura, Guzhanuer Ailiken, Mamoru Satoh, Toshinari Minamoto, Nobuko Tanaka, Fumio Nomura, Kazuyuki Matsushita

**Affiliations:** ^1^ Department of Medical Technology and Sciences, Narita School of Health Sciences, International University of Health and Welfare, Chiba-ken, Japan; ^2^ Department of Molecular Diagnosis, Graduate School of Medicine, Chiba University, Chiba, Japan; ^3^ Division of Laboratory Medicine, Chiba University Hospital, Chiba, Japan; ^4^ Division of Clinical Mass Spectrometry and Clinical Genetics, Chiba University Hospital, Chiba, Japan; ^5^ Division of Translational and Clinical Oncology and Surgical Oncology, Cancer Research Institute, Kanazawa University and Hospital, Kanazawa, Japan

**Keywords:** FUSE-binding protein-interacting repressor, LC-MS/MS, pyruvate kinase M2, six-plex tandem mass tags labeling, thymic lymphoma

## Abstract

The switch of pyruvate kinase (PK) M1 to PKM2 is pivotal for glucose metabolism in cancers. The PKM1/M2 shift is controlled by the alternative splicing of two mutually exclusive exons in the PKM gene. PKM1 is expressed in differentiated tissues, whereas PKM2 is expressed in cancer tissues. This study revealed that the haplodeficiency of FUSE-binding protein (FBP)-interacting repressor (FIR), a transcriptional repressor of the *c-myc* gene, contributed to the splicing of PKM1 to PKM2 in mice thymic lymphoma and/or T-cell type acute lymphoblastic leukemia (T-ALL) using six-plex tandem mass tag (TMT) quantitative proteomic analysis. TMT revealed 648 proteins that were up- or downregulated in mice thymic lymphoma tissues compared with wild type mouse. These proteins included transcription factors and proteins involved in DNA damage repair, DNA replication, T-cell activation/proliferation, apoptosis, etc. Among them, PKM2 protein, but not PKM1, was upregulated in the thymic lymphoma as well as T-ALL. Using qRT-PCR, we revealed that the activation of PKM2 mRNA was higher in thymic lymphoma cells of *FIR^+/−^TP53^−/−^* mice than that in control lymphocytes of *FIR^+/+^TP53^−/−^* sorted by flow cytometry. *FIR* knockdown by siRNA suppressed hnRNPA1 expression in HeLa cells. These results indicated that FIR haplodeficiency contributes the alternative splicing of PKM1 to PKM2 by partly inhibiting hnRNPA1 expression in the thymic lymphoma cells prior to T-ALL. Taken together, our findings suggest that FIR and its related spliceosomes are potential therapeutic targets for cancers, including T-ALL.

## INTRODUCTION

Pyruvate kinase (PK) M2 is activated in cancers through alternative splicing. PKM2 activation supports aerobic glycolysis: the so-called “Warburg effect” [1]. The PKM1/M2 splicing switch is affected by many factors, including c-Myc [2]; however, the mechanism of the PKM1 switch to PKM2 in carcinogenesis is largely unclear. This study revealed that the *c-myc* regulator FBP-interacting receptor (FIR) affects the splicing of PKM1 to PKM2, at least in part, in mice, as shown by six-plex tandem mass tag (TMT) quantitative proteomic analysis. c-Myc is a transcription factor that has several functions, including cell proliferation, differentiation, tumorigenesis, apoptosis, and cell cycle control [3]. The activation of c-Myc is frequently observed in many malignancies and places a burden on RNA and protein synthesis as well as affects metabolism even during pro-tumor stages [4]. Myc-stress in particular may affect alternative splicing through spliceosome complexes in cancers [4, 5]; therefore, core spliceosomes are a promising target for the treatment of c-Myc-driven cancers [4, 5, 6]. FIR is a *c-myc* gene transcriptional suppressor and binds to FUSE-binding protein (FBP), which is a transcription factor essential for the *c-myc* gene. FIR represses *c-myc* gene transcription by suppressing TFIIH/p89/XPB helicase [7]. FIR⊿exon2 is an alternatively spliced variant of FIR that lacks exon2, which includes the transcriptional repression domain [8]. FIR⊿exon2 was recently reported to be activated in different cancers and functions as a dominant negative form of FIR, potentially inducing c-Myc in colorectal cancer tissues [8]. In addition, disturbed alternative splicing of FIR increased *c-myc* gene transcription [9] in hepatocellular carcinoma [10] and non-small cell lung cancer [11]. FIR has also been co-immunoprecipitated with spliceosome complex proteins, including SAP155 (SF3B1) and hnRNPA1 [12], whereas SAP155 is required for pre-mRNA splicing of FIR [12, 13]. Mutations of SAP155 are associated with various cancers, including hematopoietic malignancies [14], whereas *FIR^+/−^TP53^−/−^* mice generated thymic lymphoma/T-call type acute lymphoblastic leukemia (T-ALL) [15]. In this study, comparative protein profiles were examined in thymic lymphomas of *FIR^+/−^TP53^−/−^* mice by six-plex TMT analysis to investigate the molecular mechanisms of tumor development. TMT is a useful gel-free tool for comparative quantitative proteomics and biomarker identification in body fluids such as sera and gingival fluid [17–19]. Notably, FIR haplodeficiency promoted the splicing of PKM1 to PKM2 in mice thymic lymphoma without circulating tumor cells/bone marrow invasion revealed by flow cytometry analysis through potentially inhibiting hnRNPA1 expression.

## RESULTS

### Proteins preparation for TMT analysis of FIR^+/−^TP53^−/−^ mouse thymic lymphoma tissues

**Figure 1:**
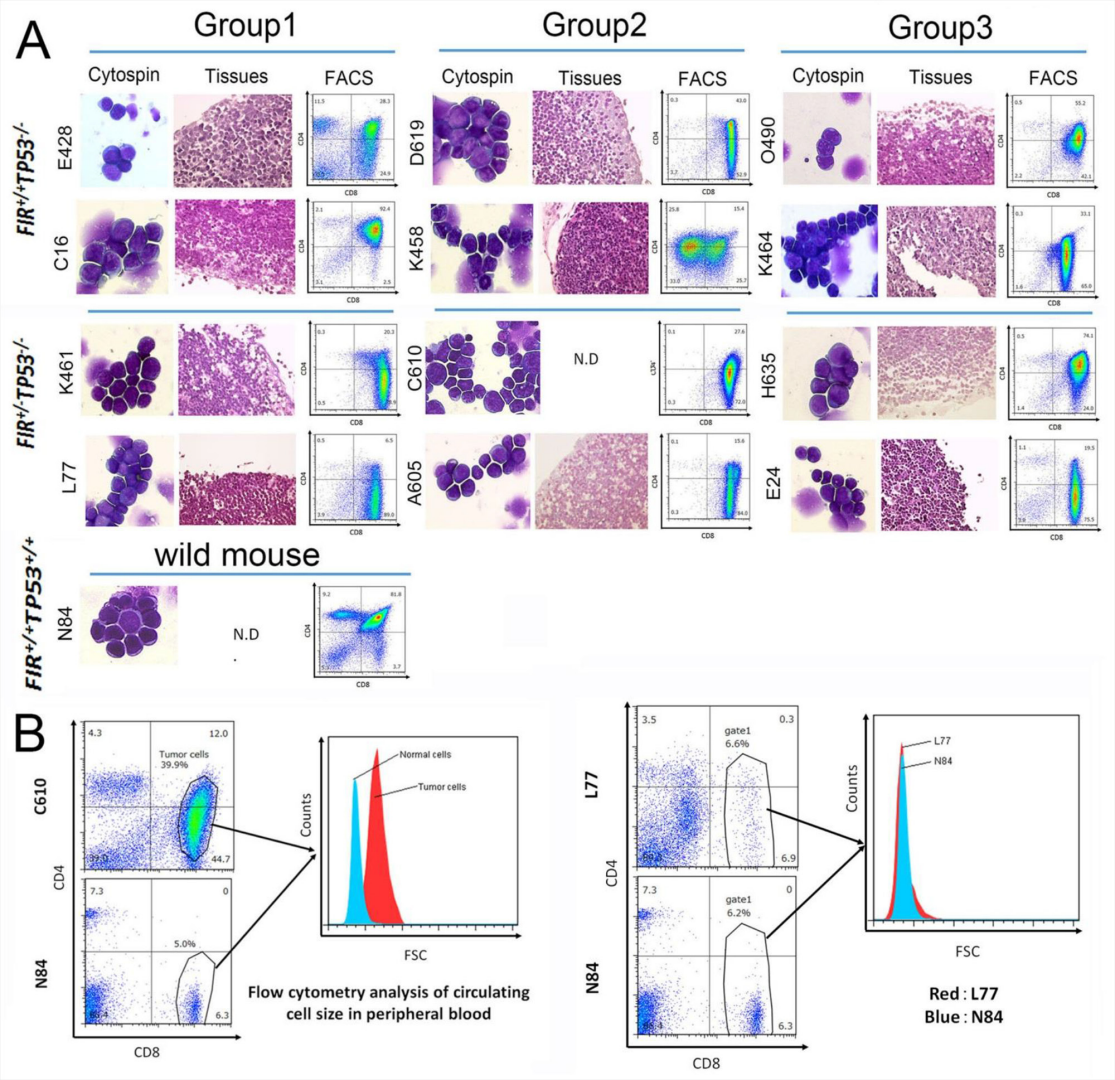
Features of thymic lymphoma in *FIR*^+/+^ *TP53*^−/−^ and *FIR*^+/−^ *TP53*^−/−^ groups using TMT analysis **(A)** Histologic features of thymic lymphoma in *FIR*^+/+^ *TP53*^−/−^ (E428, C16, D619, K458, O490, and K464) and *FIR*^+/−^ *TP53*^−/−^ (K461, L77, C610, A605, H635 and E24). Cytospin preparation and flow cytometry analysis of thymic lymphoma cells and hematoxylin and eosin (H&E) staining of paraffin-embedded thymic lymphoma tissues. TMT analyses were performed in three groups. **(B)** Flow cytometry analysis indicated the average circulating cell size by FSC (Forward scatter) at x-axis. The average circulating cell size of T-ALL mouse (C610) was larger but that of thymic lymoma mouse (L77) was as same as that of normal mouse (N84), indicated by gated area.

**Figure 2:**
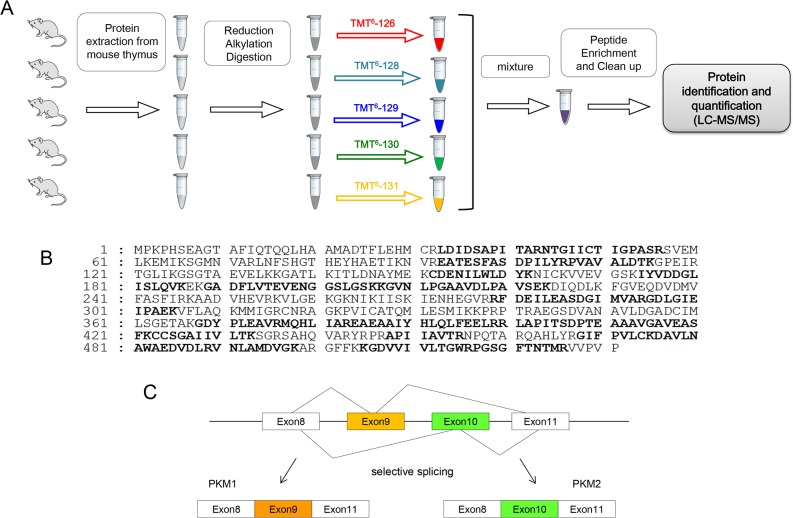
**(A)** Schematic diagram of experimental protocol. **(B)** Identification of PKM2 by LC-MS/MS. The amino acid sequence of PKM2 is shown. Matching peptide sequences are printed in bold. **(C)** Schematic diagram of PKM splicing.

Thirteen thymic lymphoma tissues, (one *FIR^+/+^TP53^+/+^*, six *FIR^+/+^TP53^−/−^*, and six *FIR^+/−^TP53^−/−^*) were used for six-plex TMT analysis (Table 1). Histopathologic and flow cytometry of tumor cells were indicate (Figure 1A). Cytospin preparation and flow cytometry analysis revealed that more than 10% of T-ALL cells were observed in bone marrows of two *FIR^+/−^TP53^−/−^* mice and one *FIR^+/+^TP53^−/−^* mouse (D619, C610 and H635 in Table 1, Figure 1A). Mice were diagnosed as thymic lymphoma in case no circulating tumor cells were detected whereas T-ALL with circulating tumor cells revealed by flow cytometry analysis (Figure 1B, Supplementary Figure 1). Six different samples can be compared in a single experiment using TMT to identify and quantity proteins by MS/MS. Samples were divided into three groups for TMT analysis. Six samples can be labeled simultaneously in our TMT protocol, therefore six *FIR^+/+^TP53^−/−^* and *FIR^+/−^TP53^−/−^*samples were divided into three groups of two. Normal thymus tissue samples from *FIR^+/+^TP53^+/+^* mice were analyzed as internal controls (Table 1, Figure 2A).

**Table 1:** Characteristics of the mice used in this study

	Mouse no.	Genotype	Phenotype	Surface marker	TMT label reagent	Thymus weight (g)	Bone marrow invasion (%)
**Wild**	N84	*FIR^+/+^TP53^+/+^*	Wild	—	TMT^6^-126	0.07	N.D.
**Group 1**	E428	*FIR^+/+^ TP53^−/−^*	Thymic lymphoma	CD4^low+^/CD8^+^	TMT^6^-128	0.96	1.1
	C16		Leukemia/thymic lymphoma	CD4^+^/CD8^+^	TMT^6^-129	0.71	N.D.
	K461	*FIR+/− TP53^−/−^*	Thymic lymphoma	CD4^low+^/CD8^+^	TMT^6^-130	0.80	N.D.
	L77		Thymic lymphoma	CD4^low+^/CD8^+^	TMT^6^-131	1.17	N.D.
**Group 2**	D619	*FIR^+/+^ TP53^−/−^*	Leukemia/thymic lymphoma	CD4^low+^/CD8^+^	TMT^6^-128	1.12	61.2
	K458		Leukemia/thymic lymphoma	CD4^+^/CD8^low+^	TMT^6^-129	0.29	5.0
	C610	*FIR+/− TP53^−/−^*	Leukemia/thymic lymphoma	CD4^low+^/CD8^+^	TMT^6^-130	1.20	42.6
	A605		Leukemia/thymic lymphoma	CD4^low+^/CD8^+^	TMT^6^-131	0.90	N.D.
**Group 3**	O490	*FIR^+/+^ TP53^−/−^*	Leukemia/thymic lymphoma	CD4^+^/CD8^+^	TMT^6^-128	0.26	N.D.
	K464		Thymic lymphoma	CD4^low+^/CD8^+^	TMT^6^-129	0.88	6.7
	H635	*FIR+/− TP53^−/−^*	Leukemia/thymic lymphoma	CD4^+^/CD8^+^	TMT^6^-130	1.01	22.6
	E24		Thymic lymphoma	CD4^low+^/CD8^+^	TMT^6^-131	1.57	N.D.

One *FIR^+/+^TP53^+/+^* sample was labeled with reporter ions at m/z = 126, two *FIR^+/+^TP53^−/−^* samples were labeled with m/z = 127, 128, and two *FIR^+/−^TP53^−/−^* samples were labeled with m/z = 129, 130, respectively (Figure 2A). Proteomic analysis of these three groups revealed 1098, 1074, 1052 proteins, respectively, 648 of which were common (Supplementary Table 3). Of these, 45 proteins were quantified 1.5 times higher/lower in *FIR^+/−^TP53^−/−^* samples compared with normal *FIR^+/+^TP53^+/+^* (Table 2, Supplementary Table 4). Identified proteins were involved in several functions, including transcriptional control, DNA repair, DNA replication, T-cell activation, T-cell proliferation, and apoptosis induction. Furthermore, hemoglobin, serum albumin, and keratin samples from the mice were included (Table 2). Five proteins were up/downregulated differently, and they included hemopexin, 60S ribosomal protein L29, fibronectin, spermidine synthase, and keratin, type II cytoskeletal 1.

**Table 2:** Up- or downregulated proteins identified by LC-MS/MS in T-ALL mice (*FIR^+/−^TP53^−/−^* and *FIR^+/+^TP53^−/−^*) compared to wild type mice (*FIR^+/+^TP53^+/+^*)

		Accession	Description	# Amino acids	MW [kDa]	T-ALL mice / wild type	Number of samples examined by TMT	
							More than 1.5 times-upregulated	Less than 0.67 times-downregulated
1	↑	P12265	Beta-glucuronidase	648	74.2	2.19	12	0
2	↑↓	Q91×72	Hemopexin	460	51.3	2.07	10	1
3	↑	Q9DCA5	Ribosome biogenesis protein BRX1 homolog	353	41.2	1.92	9	0
4	↑	P47911	60S ribosomal protein L6	296	33.5	1.86	10	0
5	↑↓	P47915	60S ribosomal protein L29	160	17.6	1.77	8	1
6	↑	P52480	Pyruvate kinase isozymes M1/M2	531	57.8	1.75	10	0
7	↑↓	P11276	Fibronectin	2477	272.3	1.53	5	1
8	↑	Q6ZQL4	WD repeat-containing protein 43	677	75.3	1.52	5	0
9	↑↓	Q64674	Spermidine synthase	302	34.0	1.34	5	3
10	↓	P07724	Serum albumin	608	68.6	1.05	2	2
11	↓	P16045	Galectin-1	135	14.9	0.74	0	6
12	↓	Q00896	Alpha-1-antitrypsin 1-3	412	45.8	0.73	1	6
13	↓	Q05816	Fatty acid-binding protein, epidermal	135	15.1	0.69	0	7
14	↓	Q61233	Plastin-2	627	70.1	0.68	0	7
15	↓	P51885	Lumican	338	38.2	0.68	0	5
16	↑↓	P04104	Keratin, type II cytoskeletal 1	637	65.6	0.68	1	7
17	↓	Q64105	Sepiapterin reductase	261	27.9	0.62	0	9
18	↓	O88531	Palmitoyl-protein thioesterase 1	306	34.5	0.62	0	7
19	↓	Q8CGP6	Histone H2A type 1-H	128	13.9	0.61	0	7
20	↓	Q9EPB4	Apoptosis-associated speck-like protein containing a CARD	193	21.4	0.57	0	9
21	↓	Q60611	DNA-binding protein SATB1	764	85.8	0.54	0	11
22	↓	P30681	High mobility group protein B2	210	24.1	0.54	0	10
23	↓	P01896	H-2 class I histocompatibility antigen, alpha chain (Fragment)	185	20.4	0.52	0	11
24	↓	Q61599	Rho GDP-dissociation inhibitor 2	200	22.8	0.52	0	10
25	↓	P99029	Peroxiredoxin-5, mitochondrial	210	21.9	0.51	0	11
26	↓	P07356	Annexin A2	339	38.7	0.51	0	10
27	↓	P01942	Hemoglobin subunit alpha	142	15.1	0.51	0	9
28	↓	P01831	Thy-1 membrane glycoprotein	162	18.1	0.50	0	9
29	↓	P02088	Hemoglobin subunit beta-1	147	15.8	0.50	0	8
30	↓	P48036	Annexin A5	319	35.7	0.50	0	12
31	↓	P68033	Actin, alpha cardiac muscle 1	377	42.0	0.50	0	10
32	↓	Q922U2	Keratin, type II cytoskeletal 5	580	61.7	0.49	0	10
33	↓	P28654	Decorin	354	39.8	0.44	0	10
34	↓	O89053	Coronin-1A	461	51.0	0.41	0	12
35	↓	P16125	L-lactate dehydrogenase B chain	334	36.5	0.38	0	12
36	↓	P15864	Histone H1.2	212	21.3	0.38	0	12
37	↓	P02089	Hemoglobin subunit beta-2	147	15.9	0.27	0	12
38	↓	P02301	Histone H3.3C	136	15.3	0.27	0	12
39	↓	P62806	Histone H4	103	11.4	0.26	0	12
40	↓	Q62426	Cystatin-B	98	11.0	0.22	0	12
41	↓	P08074	Carbonyl reductase [NADPH] 2	244	25.9	0.18	0	12
42	↓	Q9QWL7	Keratin, type I cytoskeletal 17	433	48.1	0.18	0	12
43	↓	Q9DB60	Prostamide/prostaglandin F synthase	201	21.7	0.15	0	12
44	↓	P11679	Keratin, type II cytoskeletal 8	490	54.5	0.11	0	12
45	↓	P05784	Keratin, type I cytoskeletal 18	423	47.5	0.08	0	12

Details of each sample are listed in Supplementary Table 4

### PKM2 was activated at protein and mRNA levels in thymic lymphoma of FIR^+/−^TP53^−/−^ mice

**Figure 3:**
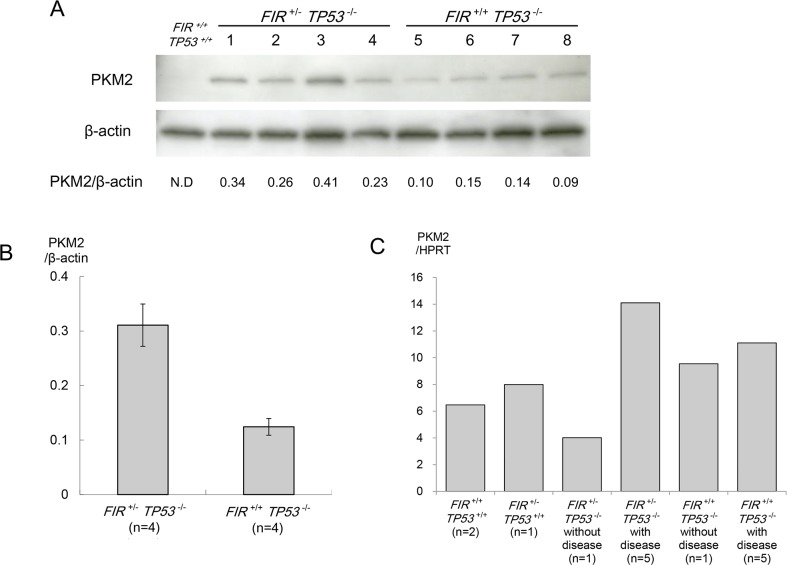
Western blot analysis of pyruvate kinase (PK) M2 **(A)** Proteins extracted from each mouse thymus were separated by 10.0–12.0% SDS-PAGE and probed with anti-PKM2. Expression levels are relatively higher in *FIR*^+/−^ *TP53*^−/−^ samples than *FIR*^+/+^ *TP53*^−/−^. **(B)** Differences in expression were analyzed using the Student's t-test. The expression levels of these proteins were upregulated significantly in *FIR*^+/−^ *TP53*^−/−^ group (p = 0.002). Band intensities were quantified by image analysis software. The line indicates the range and the rectangle indicates the mean values. **(C)** *PKM2* mRNA expression was significantly upregulated in thymic lymphoma of *FIR*^+/−^*TP53*^−/−^ mice.

PKM2 protein was upregulated in the thymic lymphoma of *FIR^+/−^TP53^−/−^* mice compared with *FIR^+/+^TP53^−/−^* mice (Figure 2B, 2C). Western blotting confirmed that PKM2 was overexpressed 2.5 fold in the thymic lymphoma of *FIR^+/−^TP53^−/−^* mice compared with *FIR^+/+^TP53^−/−^* mice (p = 0.002) (Figure 3A, 3B), while *PKM2* mRNA was elevated approximately twice more in the thymic lymphoma of *FIR^+/−^TP53^−/−^* mice than the *FIR^+/+^TP53^−/−^* mice (Figure 3C). These results indicated that *FIR* haplodeficiency promotes splicing of PKM1 to PKM2 in benign thymic lymphoma at the transcriptional level.

### Knockdown of FIR suppressed hnRNPA1 expression

**Figure 4:**
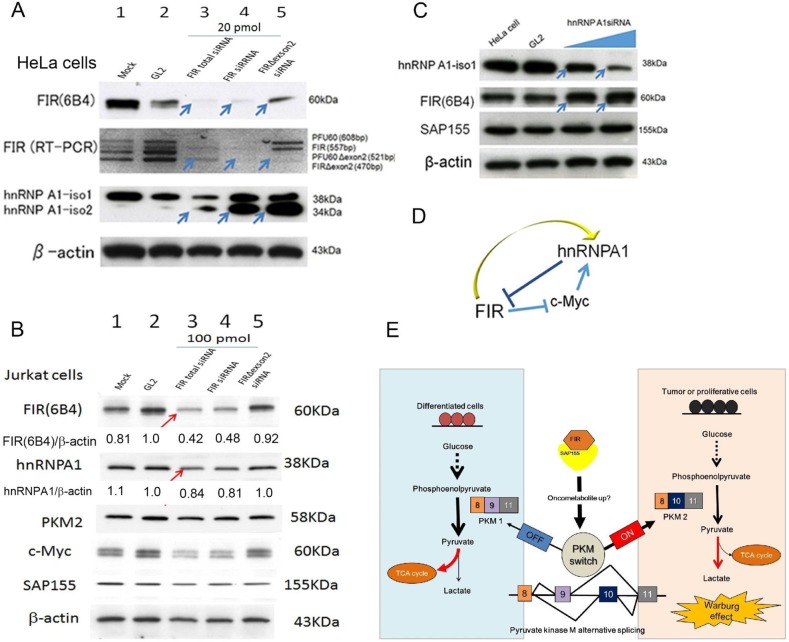
Knockdown of *FIR* suppressed hnRNPA1 expression FIR and hnRNPA1 expression was examined after siRNA treatment of HeLa cells. *GL2* siRNA was transfected as the negative control. After 48 h and 72 h of incubation, whole-cell extracts were analyzed by western blotting. **(A)** Three different types of *FIR* siRNAs were transfected into HeLa cells. Lane 2 is the *GL2* siRNA control transfection, lane 3 is 20 pmol of FIR total (FIR and FIRΔexon2) siRNA, lane 4 is 20 pmol FIR-specific siRNA transfection, lane 5 is 20 pmol FIRΔexon2-specific siRNA. HnRNPA1 expression was down-regulated by total FIR siRNAs (Lane 3 as compared to Lanes 1, 2, arrows). **(B)** Three different types of *FIR* siRNAs were transfected into Jurkat cells. Lane 2 is the *GL2* siRNA control transfection, lane 3 is 100 pmol of FIR total (FIR and FIRΔexon2) siRNA, lane 4 is 100 pmol FIR-specific siRNA transfection, lane 5 is 100 pmol FIRΔexon2-specific siRNA. HnRNPA1 expression was down-regulated by total FIR siRNAs (Lane 3 as compared to Lanes 1, 2, arrows). **(C)** HnRNPA1 siRNAs were transfected into HeLa cells in dose-dependent manner. Lane 2 is *GL2* siRNA, lane 3 is 20 pmol type V1 hnRNPA1, hnRNPA1 siRNA, and lane 4 is 20 pmol type G5 hnRNPA1 siRNA. FIR expression was upregulated by hnRNPA1 siRNA (arrows). **(D)** Schematic view of the relationship among FIR, hnRNPA1 and c-Myc. **(E)** *FIR* haplodeficiency promotes splicing to PKM2 through affecting alternative splicing switch in mice thymic lymphoma tissues using TMT analysis.

Haplodeficiency of *FIR* upregulated PKM2 at protein and mRNA levels (Figure 2B, 2C), and HnRNPA1 promotes splicing of PKM1 to PKM2 [21, 22]. Notably, FIR was co-immunoprecipitated with hnRNPA1 in nuclear extracts of HeLa cells [12], suggesting that FIR affects the ratio of PKM1:PKM2 via hnRNPA1. Based on these results, we investigated the relationship between FIR and hnRNPA1. Knockdown of *FIR* by siRNA suppressed hnRNPA1 expression in HeLa cells (Figure 4A, arrows) and Jurkat cells (Figure 4B, arrows), indicating that altered FIR expression affected PKM1/PKM2 splicing through hnRNPA1 expression. *FIR* haplodeficiency activates c-Myc in mice [15], which itself directly induces hnRNPA1 [23]; therefore FIR-mediated hnRNPA1 suppression requires c-Myc. Further study is required to elucidate this feedback mechanism (Figure 4C, 4D). In conclusion, *FIR* haplodeficiency switches PKM1 to PKM2 in thymic lymphoma, possibly by affecting hnRNPA1 expression (Figure 4E).

## DISCUSSION

*FIR^+/−^TP53^−/−^ and FIR^+/+^TP53^−/−^* mice that develop thymic lymphoma and T-ALL were generated previously [15]. We performed proteomic and qRT-PCR analyses in thymic lymphoma tissues and revealed that PKM2 was significantly higher at both mRNA and protein levels in thymic lymphoma of *FIR^+/−^TP53^−/−^* mice than in *FIR^+/+^TP53^−/−^* mice. This indicates that FIR haplodeficiency switches PKM1 to PKM2 at the transcriptional level, *FIR* knockdown suppressed hnRNPA1 expression, and hnRNPA1 promotes splicing of PKM1 to PKM2 and was reported to be coimmunoprecipitaed with FIR [12]. This study performed the requisite statistical analysis to make an argument that FIR and its related spliceosomes are potential therapeutic targets for cancers, including T-ALL.

TMT quantitative analysis is a gel-free proteomic technique and can be used to analyze various samples, including sera/plasma, body fluids, or gingival crevicular fluids [17–19]. Using this approach, we examined the altered protein expression profile of thymic lymphoma tissues under *FIR* haplodeficient conditions. In addition, we revealed that FIR/FIRΔexon2 was co-immunoprecipitated with PTB, hnRNP L/M, and PCBP1, which are known splicing factors of CD44 [12]. This suggested that altered FIR expression affects PKM1/PKM2 splicing by modifying hnRNPA1 expression. Moreover, *FIR* haplodeficiency activates c-Myc in thymic lymphoma cells in mice [15]. c-Myc directly induces hnRNPA1 [23], indicating that hnRNPA1 suppression by *FIR* siRNA depends partly on the c-Myc pathway. Further study is required to elucidate this feedback mechanism.

FIR links *c-myc* transcriptional regulation and alternative splicing [13]. *FIR* variants are activated in colorectal cancers [8], hepatoma [10], non-small cell lung cancer [11], and leukemia [15]. FIR is also called as PUF60, which is a member of U2AF splicing factor family [24]. An alternatively spliced dominant negative form of FIR is activated in cancer tissues. Notably, FIR forms a complex with SAP155 (SF3B1), which is frequently mutated in hematopoietic malignancies [25] and affects alternative splicing [14]. The FIR-SAP155 (SF3B1) interaction affects both hnRNPA1 and c-Myc expression and may represent a therapeutic target in c-Myc-driven cancer.

Normal differentiated cells depend on mitochondrial oxidative phosphorylation to generate energy. In cancer cells, energy is produced by aerobic glycolysis, called “the Warburg effect” [1]. Switching from PKM1 to PKM2 promotes aerobic glycolysis and tumor formation [23]. This study revealed that the switch from PKM1 to PKM2 occurs in lymphoma cells prior to T-ALL, indicating that PKM2 activation is required but not sufficient for T-ALL progression in this mouse model. Moreover, *FIR* haplodeficiency potentially affects PKM1/PKM2 splicing by modifying hnRNPA1 expression.

In the case of tumors originating from differentiated cells, high levels of PKM2 in tumor or cancer initiating cells are attributed to the switch of PKM gene splicing from PKM1 to PKM2. In contrast, PKM2 expression is directly inherited from the precursor/stem cells [26]. Our results support the notion above that PKM2 is activated in non-malignant thymic lymphoma. Therefore, FIR is a potential target for cancer therapy. Because the function of PKM2 in tumor progression seems to be regulated in multiple ways [26], further studies are required to fully elucidate this complicated mechanism.

## MATERIALS AND METHODS

### Preparation of genetically engineered mice tissues

Genetically manipulated mice were generated as previously described [15]. Thymus lymphoma and/or T-ALL was generated in *FIR^++^TP53^+/+^* and *FIR^+/−^TP53^−/−^* mice as previously described [15] and thymus tissues were extracted. Details of all samples are described in Table 1. Mice with similar phenotypes were divided into groups for the quantitative protein assay and *FIR^++^TP53^+/+^* mice were used as controls.

### Cancer cell lines

HeLa and Jurkat cell lines (acute T-cell leukemia cells) were cultured in IMDM and RPMI 1640 (ThermoFisher Scientific Diagnostics CO., LTD, Tokyo, Japan), respectively, supplemented with 10% FBS and 1% penicillin–streptomycin. Cells were grown at 37°C in a 5% CO_2_ incubator.

### Western blotting and antibodies for cancer cell lines

Culture medium was removed, and cells were washed twice with cold (4°C) PBS. Then, cells were lysed with 1:20 β-mercaptoethanol in 2X sample buffer and incubated at 100°C for 5 min. Whole-cell lysates were assayed for protein content (Bio-Rad, Hercules, CA, USA), and 10 μg of proteins were separated by SDS-PAGE on 7.5% or 10–20% XV PANTERA gels before being transferred onto polyvinylidene fluoride membranes using a tank transfer apparatus. The membranes were blocked with 0.5% skimmed milk in PBS overnight at 4°C. Antigens were detected with enhanced chemiluminescence detection reagents (GE Healthcare UK Ltd., Buckinghamshire, UK). Membranes were incubated with primary antibodies for 1 h at room temperature, followed by three 10-min washes with 1XPBS/0.01% Tween 20. Membranes were then incubated with commercial secondary antibodies, followed by three 15-min washes with 1XPBS/0.01% Tween 20. The primary mouse monoclonal antibody against the FIR C-terminus (Total FIRs 6B4) was prepared by Dr. Nozaki [16].

### Flow cytometry and cell sorting

Flow cytometry and cell sorting were performed as described previously [15]. Thymic lymphoma cells were stained with APC-conjugated CD4, PE-conjugated CD8α and Pacific Blue-conjugated CD45.2 (BioLegend). Dead cells were detected by staining with 1 μg/mL propidium iodide (Sigma-Aldrich), and flow cytometry and cell sorting were performed on a FACSCanto II or FACSAria II flow cytometer (BD Biosciences).

### Protein extraction from mice thymus

Thymi from *FIR^+/+^TP53^−/−^* and *FIR^+/−^TP53^−/−^* mice were frozen in liquid nitrogen and then floated in 20X 4 M urea/100 mM ammonium bicarbonate. Floating pieces of thymus tissue were homogenized and centrifuged at 24,000 rpm at room temperature using a POLYTRON^®^ homogenizer (KINEMATICA, Schweiz). After centrifugation, thymus cells were completely disrupted by sonication three times for 10 sec each, and after homogenization and sonication, samples were centrifuged at 50,000 rpm at 4°C for 60 min before the supernatant was collected. Extracted protein supernatant samples were stored at −80°C before protein digestion.

### Protein in-solution digestion

Extracted samples of 100 μg protein were mixed with 100 μL of 4 M urea/100 mM ammonium bicarbonate after measuring protein concentrations. Then, 100 μL of 4 M urea/100 mM ammonium bicarbonate, including 2% 200 mM DTT, was added and incubated at 57°C for 30 min. After incubation, 10 μL of 600 mM iodacetoamide was added and incubated at room temperature for 30 min in the dark. Twenty microliters of lysyl endopeptidase were incubated in 200 μL of distilled water at 37°C for 3 h, and then, 25 μL of trypsin were incubated with 200 μL of distilled water overnight at 37°C. The resulting peptides were added to 50 μL of 10% TFA.

### Protein extraction, reduction and TMT labeling

After protein extraction and reduction using Stage Tip (Thermo Fisher Scientific Inc, USA), samples were digested and lyophilized before being resuspended in 40 μL of 0.1% TFA. After vortexing at room temperature for 5 min, 40 μL of triethyl ammonium bicarbonate were added and mixed. An Isobaric Label Reagent set (Pierce, Idaho, ID, USA) was used for six-plex TMT according to the manufacturer's instructions. Each mass-tagging reagent tube contained 0.8 mg of different isobaric chemical tags with 42 μL of anhydrous acetonitrile, dissolved for 5 minutes with occasional vortexing and brief centrifugation to gather the solution. Samples were then incubated in 42 μL of the unique reagent for 1 h at room temperature. The reaction was quenched by incubation in 8 μL of 5% hydroxylamine for 30 min. Samples were cleaned using C18 spin tips, and stored at −80°C until LC-MS/MS analysis (Figure 2A).

### LC-MS/MS

Enriched digested peptides were injected into a trap column (C18, 0.3×5 mm, DIONEX, CA, USA) and analytical column (monolithic silica-C18, 0.1×2000 mm, GL sciences, Tokyo, Japan), which was attached to the Ultimate 3000 (DIONEX, CA, USA).

The flow rate of the mobile phase was 500 nL/min, with a solvent composition programmed to change in 480 min cycles with varying ratios of solvent A (2% v/v CH3CN and 0.1% v/v HCOOH) to solvent B (90% v/v CH3CN and 0.1% v/v HCOOH) as follows; 5–10% B 60 min, 10–30% B 280 min, 30–40 % B 60 min, 40–100% B 2 min, 100% B 5 min, 100–5% B 2 min, and 5% B 71 min. Purified peptides from HPLC were introduced to a LTQ-Orbitrap XL hybridion-trap Fourier transform mass spectrometer (Thermo Scientific, San Jose, CA, USA) using a Pico Tip (New Objective, MA, USA). A cycle scan event included one full scan and three data-dependent, CID, high-energy, collision-activated, dissociation dual MS/MS scans for TMT reporter ions [19]. The database search engine (Proteome discoverer; version 1.3.0, Thermo Scientific, San Jose, CA, USA) was used to identify proteins based on mass, tandem mass spectra, and reporter ion spectra of peptides. Peptide mass data were matched by searching the UniProtKB Mouse database (SwissProt 2014, 16951 entries), using the following search parameters: peptide mass tolerance, 2 ppm; fragment tolerance, 0.6 Da; enzyme set to trypsin, allowing up to two missed cleavages; dynamic modifications, methionine oxidation; static modifications, cysteine carbamidomethylation, and; TMT tagged N-terminus and lysine. The minimum criteria of protein identification were filtered with Xcorr versus charge state and set as false discovery rate (FDR) < 1%. The FDR was estimated by comparing with a randomized decoy database created by the Proteome Discoverer 1.3.0 program supplied by Thermo Scientific.

### Quantitative real-time PCR (qRT-PCR)

Quantitative real-time PCR to measure *PKM2* was performed as previously described [9, 12] (Supplementary Table 1).

### Western blotting

Details of primary and secondary antibodies are given in Supplementary Table 2A. Briefly, 20 μg mouse thymus protein extracts were separated using SDS polyacrylamide gel electrophoresis and transferred to a PVDF membrane (Millipore, Bedford, MA) as described previously [20]. The membranes were incubated with anti-PKM2 antibody (ab154816; rabbit monoclonal, Abcam, Cambridge, MA, USA) at 1:200 dilutions for 60 min at room temperature. After primary antibody incubation, membranes were washed three times for 30 min in PBS-Tween (0.1%), prior to incubation in the appropriate horseradish peroxidase-linked secondary antibody (goat anti-rabbit IgG horseradish peroxidase-linked antiserum) diluted 1:2500 for 60 min at room temperature. Finally, membranes were washed three times as previously described, and immunoreactive proteins were revealed with an enhanced chemiluminescence substrate reaction using Pierce® ECL Plus western blotting substrate (Thermo Scientific, San Jose, CA, USA) according to the manufacturer's instructions. Total-Lab TL12 imaging analysis software (Shimadzu Co., Ltd. Kyoto, Japan) was used for quantification of bands intensities and statistical analysis.

### FIR and hnRNPA1 siRNA

Three *FIR* siRNAs and two *hnRNPA1* siRNAs were purchased from Sigma-Aldrich (Supplementary Table 2B). siRNAs were transiently transfected using Lipofectamine 2000 (Invitrogen) for HeLa cells and HiPerFect Transfection Reagent (QIAGEN) for Jurkat cells according to the manufacturer's instructions. After transfection with *FIR* siRNA, cells were cultured for 48 h and after transfection with *hnRNPA1* siRNAs, cells were cultured for 72 h, at 37°C in a CO_2_ incubator.

### Statistical analysis

Numerical data were presented as mean ± standard deviation (SD). Statistical significance was assessed using the Student's t-test and P values less than 0.05 were considered statistically significant.

## SUPPLEMENTARY MATERIALS FIGURE AND TABLES











## Abbreviations

PK: pyruvate kinase
FBP: FUSE-binding protein
FIR: FBP-interacting repressor
T-ALL: T-cell type acute lymphoblastic leukemia
TMT: six-plex tandem mass tag
