# Supplementary material for: FIR haplodeficiency promotes splicing to pyruvate kinase M2 in mice thymic lymphoma tissues revealed by six-plex tandem mass tag quantitative proteomic analysis
Source: Oncotarget. 2017 Jul 7;8(40):67955–65. doi: 10.18632/oncotarget.19061 (PMC5620227; doi:10.18632/oncotarget.19061)
Supplement: Supplementary file 1 [file oncotarget-08-67955-s001.pdf]

FIR haplodeficiency promotes splicing to pyruvate kinase M2 in mice thymic lymphoma tissues revealed by six-plex tandem mass tag quantitative proteomic analysis

SUPPLEMENTARY MATERIALS

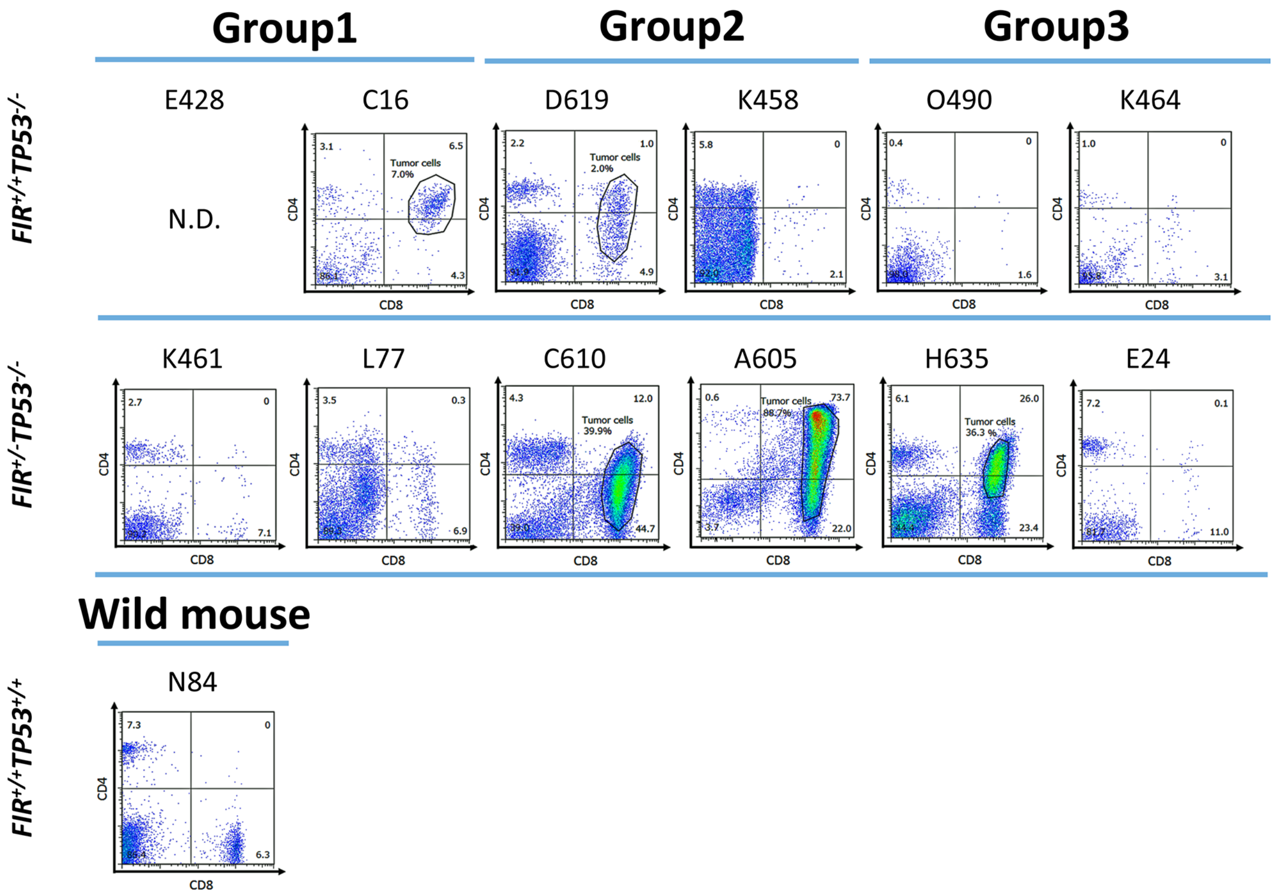

Supplementary Figure 1: Features of circulating tumor cells in *FIR*$^{+/-}$ *TP53*$^{-/-}$ and *FIR*$^{+/+}$ *TP53*$^{-/-}$ groups. Mice were diagnosed as thymic lymphoma in case no circulating tumor cells (K461, L77, K464 and E24) were detected whereas T-ALL with circulating tumor cells (C16, D619, K458, C610, A605 and H635) revealed by flow cytometry analysis. E428 and O490 were checked by peripheral blood smear. C610 was reported as described previously [15].

Supplementary Table 1: Primers and PCR conditions used in this study

See Supplementary File 1

Supplementary Table 2A: Details of primary and secondary antibodies used in this study. Supplementary Table 2B: Primers, siRNAs and PCR conditions used in this study

See Supplementary File 2

Supplementary Table 3: Proteins revealed by proteomic analysis using six-plex TMT labeling

See Supplementary File 3

Supplementary Table 4: Up- or downregulated proteins which commonly reveraled in three groups identified by LC-MS/MS in T-ALL mice (*FIR*$^{+/-}$*TP53*$^{-/-}$ *and FIR*$^{+/+}$*TP53*$^{-/-}$) compared to wild type mice (*FIR*$^{+/+}$*TP53*$^{+/+}$)

See Supplementary File 4
